# Perceived quality of essential newborn care implementation among health facility deliveries in North Gondar Zone, Northwest Ethiopia: a cross-sectional study

**DOI:** 10.1186/s12978-021-01175-y

**Published:** 2021-06-13

**Authors:** Tadesse Guadu Delele, Gashaw Andargie Biks, Solomon Mekonnen Abebe, Zemene Tigabu Kebede

**Affiliations:** 1 Department of Environmental and Occupational Health, and Safety, Institute of Public Health, College of Medicine and Health Sciences, University of Gondar, Gondar, Ethiopia; 2 Departments of Health System and Policy, Institute of Public Health, College of Medicine and Health Sciences, University of Gondar, Gondar, Ethiopia; 3 Departments of Human Nutrition, Institute of Public Health, College of Medicine and Health Sciences, University of Gondar, Gondar, Ethiopia; 4 Departments of Pediatrics and Child Health, School of Medicine, College of Medicine and Health Sciences, University of Gondar, Gondar, Ethiopia

**Keywords:** Newborn care, Essential newborn care, Quality of care

## Abstract

**Background:**

Quality of essential newborn care is defined as the extent of health care services to improve the health of newborns. However, studies are scarce regarding the quality of newborn care implementation. Therefore, this study aimed to measure the magnitude and factors associated with essential newborn care implementation perceived quality among health facility deliveries in Northwest Ethiopia.

**Methods:**

A facility-based cross-sectional study design was employed to collect data from 370 randomly selected deliveries in 11 health facilities from November 2018 to March 2019. Essential newborn care implementation perceived quality was assessed in two domains (delivery and process) from clients’ perspectives. A pre-tested interviewer-administered structured questionnaire was adopted from different kinds of literature and guidelines. The research data were collected by trained midwives and nurses. A binary logistic regression model was used to identify associated factors with newborn care implementation perceived quality. Odds ratio with 95% CI was computed to assess the strength and significant level of the association at *p*-value < 0.05.

**Results:**

About 338 mothers completed the interview with a response rate of 97.1%. The mean age of the study participants was 26.4 (SD = 5.7) with a range of 12 and 45 years. Most mothers, 84.3%, have attended antenatal care. The overall implementation perceived quality of essential newborn care was found to be 66.3%. The implementation perceived quality of cord care, breast-feeding and thermal care was 75.4, 72.2 and 66.3% respectively. Newborn immunization and vitamin K administration had the lowest implementation perceived quality i.e. 22.4 and 24.3% respectively. Friendly care during delivery (AOR = 5.1, 95% CI: 2.4, 11.0), partograph use (AOR = 3.0, 95% CI: 1.1, 8.6), child immunization service readiness (AOR = 2.9, 95% CI: 1.5, 5.7), BEmEONC service readiness (AOR = 2.1, 95% CI: 1.2, 3.9) and facing no neonatal illness at all (AOR = 4.2, 95% CI: 1.6, 10.9) were significantly associated with good essential newborn care implementation qualities.

**Conclusions:**

The perceived quality of essential newborn care implementation was low in the study area. This is associated with poor readiness on BEmEONC and child immunization services, unfriendly care and not using partograph during delivery. Hence, availing the BEmEONC and the child immunization service inputs, continuous training and motivation of healthcare workers for friendly care are vital for improving essential newborn care implementation perceived quality.

## Plain language summary

Essential newborn care is a set of practices provided by healthcare workers and mothers to every newborn during delivery. Studies are scarce regarding the quality of newborn care implementation. Therefore, this study aimed to measure the magnitude and factors associated with essential newborn care implementation perceived quality among health facility deliveries in Northwest Ethiopia.

A facility-based cross-sectional study design was employed to collect data from 370 randomly selected deliveries in 11 health facilities. A pre-tested interviewer-administered structured questionnaire was adopted from different kinds of literature and guidelines. A binary logistic regression model was fitted to assess the strength and significant level of the association at *p*-value < 0.05.

The overall implementation perceived quality of essential newborn care was found to be 66.3%. The implementation perceived quality of cord care, breast-feeding and thermal care was 75.4, 72.2 and 66.3% respectively. Newborn immunization and vitamin K administration had the lowest implementation perceived quality i.e. 22.4 and 24.3% respectively. Friendly care during delivery (AOR = 5.1, 95% CI: 2.4, 11.0), partograph uses (AOR = 3.0, 95% CI: 1.1, 8.6), child immunization service readiness (AOR = 2.9, 95% CI: 1.5, 5.7), BEmEONC service readiness (AOR = 2.1, 95% CI: 1.2, 3.9) and facing no neonatal illness at all (AOR = 4.2, 95% CI: 1.6, 10.9) were significantly associated with good essential newborn care implementation qualities.

The perceived quality of essential newborn care implementation was low in the study area. This is associated with poor readiness on BEmEONC and child immunization services, unfriendly care and not using partograph during delivery. Hence, availing the BEmEONC and child immunization service inputs, continuous training and motivation of healthcare workers for friendly care are vital for improving essential newborn care implementation perceived quality.

## Background

Ensuring the quality of newborn care during delivery is crucial to improve health outcomes and reduce preventable mortality and morbidity among newborns. Although access to institutional delivery care and the presence of skilled birth attendants have increased globally, a substantial proportion of newborns experience many avoidable deaths within health facilities due to quality issues [1]. The World Health Organization (WHO) also stated that universal health coverage (UHC) and quality of care (QoC) are now recognized as the two main pillars for addressing these preventable deaths [2].

Essential Newborn Care (ENC) is a set of measures every newborn baby needs regardless of its birthplace and size. ENC is a framework that should be applied immediately after birth and continued at least for the first seven days to protect the newborn from adverse environmental problems [3–5]. Components of ENC and neonatal resuscitation are proven interventions for reducing neonatal mortality rate and stillbirth rate [6].

Globally, deaths among children aged 1 month to 5 years old have fallen dramatically in the recent decades. However, progress in reducing the deaths of newborn babies aged less than 1 month have been less notable because 7,000 newborns are still dying every day. This is because of the difficulty to address and prevent newborn deaths with a single drug or intervention; these require a system-wide approach that improves the quality of newborn care [7].

In Ethiopia, the rate of under-five mortality (U5MR) was decreased by 55%, i.e. from 123 to 55 per 1000 live births from the year 2005 to 2019. But, the neonatal mortality rate was decreased only from 39 to 30 per 1000 live births from 2005 to 2019. Because of this, the share of neonatal mortality in under-five mortality has been increased from 31 to 55% [8]. Most neonatal health problems are life-threatening. Therefore, they need optimal care for their survival [9].

Scientific pieces of evidence have also shown that many neonatal deaths and illnesses can be prevented through evidence-based interventions, including appropriate utilization of essential newborn care packages, which require clinically trained providers [10]. A research finding [11] also estimated that a skilled birth care package could reduce neonatal mortality by 20–30%.

To combat this problem, Ethiopia emphasizes access to quality health services for all Ethiopians with full financial risk protection without any discrimination like age, economic capability, and geographic locations [12]. Though there are no comprehensive studies on the implementation quality of essential newborn care, different pocket studies in Ethiopia stated that the quality of newborn care practice was low [13–15]. Most of the previous studies mainly focused on a few components of essential newborn care services at the community level. To adopt a focused and evidence-based approach to improve essential newborn care quality in Northwest Ethiopia, a clear understanding of the current implementation quality in health facility deliveries and associated factors is necessary. Therefore, this study aimed to assess the magnitude and factors associated with essential newborn care implementation perceived quality by focusing on facility inputs and process quality components. The findings were important in planning intervention programs to improve the survival of neonates in the study area and other similar settings.

## Methods

### Study area and health facility setting

This study was conducted at health facilities in North Gondar Zone, Northwest Ethiopia. This administrative Zone is structured in eight districts, including the two town administrations. Debark town, which is the capital of the zone, is 90 and 820-kilo meters away from Gondar and Addis Ababa, respectively. According to the Central Statistical Agency (CSA), North Gondar Zone has an estimated total population of 887,869 individuals, 37 health facilities providing delivery services (2 hospitals and 35 health centers), and 1230 healthcare workers in 2016/17. More specifically, all delivery service providing public health facilities in three randomly selected districts (Dabat, Debark and Janamora) were included in the study. Sixteen public health facilities (14 health centers and 2 district hospitals) were considered for data collection [16].

### Study design and period

The facility-based cross-sectional study design was employed among selected health facility deliveries in Northwest Ethiopia from November 2018 to March 2019.

### Source and study population

All mothers in North Gondar Zone, who delivered in governmental health facilities, were the source populations. All mothers who delivered in randomly selected districts and health facilities were the study populations. And all mothers who delivered in the randomly selected districts and health facilities and who were randomly selected for inclusion were the study subjects.

### Inclusion and exclusion criteria

All mothers who gave live birth/s (having evidence of life, such as the beating of heart, pulsation of the umbilical cord, or definitive movement) in the randomly selected health facilities during the study period, and who were randomly selected for inclusions as well as willing to participate were part of the inclusion criteria. Eligible mothers who were not willing to participate, seriously ill and or unable to respond to all the assessment questions were part of the exclusion criteria.

### Measurements

**Essential newborn care** is a set of practices provided by healthcare workers and mothers to every newborn during delivery [9]. It was measured by using components and domains.

**A component** is an activity or set of activities, which are basic to newborn survival. Thirty-two question items were structured in 2 domains and 21 components. The items were prepared as YES/NO questions adopted from newborn care guidelines and different kinds of literature [4, 17–25]. Then, a composite variable from these questions was generated to categorize each component as having “Good/Poor implementation perceived quality”. Each component was categorized as good perceived quality if it scored mean and above the mean value, and poor perceived quality if otherwise.

#### Optimum thermal care

Wiped off/ dried the baby within ten minute, wrapped in new or clean and dry old cloth and washing the body of the newborn by warm water after 24 h of delivery to prevent hypothermia.

#### Safe cord care

The use of a clean cutting instrument to cut the umbilical cord (boiled new, used blade or scissor) plus clean thread, cord tie or cord clamp and no any substance applied on the cord stump.

#### Breastfeeding

Initiate breastfeeding within the first one hour after birth, giving no prelacteal and feeding the child with colostrum.

#### Eye care

The provision of tetracycline eye ointment to the newborn after birth and advising on how to maintain the newborn’s eye healthy.

#### Vitamin K administration

The provision of 1 mg Vitamin K on anterior mid-lateral thigh to prevent the newborn from bleeding.

#### Immunization

The provision of BCG and OPV0 vaccines to the newborn immediately after birth.

#### Skin to skin care

Placing the infant in skin-to-skin contact on the mother’s chest and cover both with clean linen and blanket.

#### Helping baby breath

If the baby is not breathing or gasping, then resuscitate.

#### Weighing

Weight the newborn within 90 min after birth.

#### Chlorhexidine

Apply Chlorohexidine gel (4%) on the cord within 30 min.

#### Safety practices during delivery

The delivery assistant tried to minimize/avoid all the suffering: pains, all the possible medical errors during delivery.

#### Effectiveness in using guidelines and knowledge on reproductive health

The delivery assistant helped you as per the delivery service provision guideline, based on his/her scientific knowledge skill properly during delivery.

#### Timely service provision

The health care providers provided you delivery service without delay and bureaucracy.

#### Efficiency of using resources

The delivery care provider provided the service by minimizing unnecessary delivery related resource wastages.

#### Equitability of delivery services

The health care provider provided delivery services equitably (without any discrimination; like relation, neighboring, race, social etc.).

#### Acceptability or People-centered delivery service provision

The delivery service provider provided the service based on your interest by keeping the culture and norms of the society during delivery.

#### Accessibility of delivery services

The delivery ward of the health facility was accessible for vehicle transport, and found within short distance from your home to easily utilize the service.

#### Responsiveness of health care workers

The delivery care providers provided the service immediately and properly in responsive manner.

#### Respect for customers

The delivery care providers provided the service by respecting personal dignity and personality (without harassment).

#### Hygiene and sanitation practices

The delivery care providers tried to minimize the hygienic, sanitary problems and properly manage wastes during delivery.

#### Team-work during delivery

The health care providers in the delivery ward work in team during delivery.

**A domain** includes a group of components with a set of practices provided to every newborn. Two domains; ***delivery service*** and ***process*** domains were composite variables defined for this study purpose.

**Delivery service** domain was defined as a composite variable containing 10 components. This domain measures how the delivery service was provided as per the guidelines. Thermal care, cord care, breast-feeding, eye care, vitamin K administration, and immunization were the main components under the delivery care domain. Skin to skin care, Weighing, Helping baby breath, and Chlorhexidine gel application were the other components under delivery care domain.

The process quality domain was also defined as a composite variable containing 11 component. This domain measures how the delivery service provision process of efficient enough from mothers’ perspective. The components include; safety, effectiveness, timeliness, efficiency, equitability, acceptability or people-centered, accessibility, responsiveness, respect, hygiene and sanitation, and team-work. Each domain perceived quality was good if it scored the mean and above the mean value of the sum of components in each domain, and poor perceived quality if otherwise.

**Essential newborn care implementation perceived quality** score was categorized as good if they scored the mean and above the mean value of the sum of scores in the two domains (service and process), and poor perceived quality if otherwise.

#### Item

It is a piece of question used to ascertain either the presence or absence of something. BEmEONC and Child immunization services were assessed by adopting WHO BEmEONC and Child immunization service domain items (Trained staff and guidelines, Equipment, Medicines and commodities).

#### Trained staff and guidelines

Assess the availability of trained staffs and guidelines on BEmEONC and Child immunization as per the WHO guideline.

#### Equipment

Assess the availability of different equipment applicable for BEmEONC and Child immunization services as per the WHO guideline.

#### Medicines and commodities

Assesse the availability of different medicines and commodities used for BEmEONC and Child immunization services as per the WHO guideline.

**BEmEONC service readiness** was measured using three domains (Trained staff and guidelines, Equipment, and Medicines and commodities) with 25 items. Facilities were categorized to have good readiness if it scored the mean and above the mean value of the sum of scores in all domains, and poor if otherwise.

**Child immunization service readiness** was measured using three domains (Trained staff and guidelines, Equipment, and Medicines and commodities) with 16 items. Facilities were categorized to have good readiness if it scored mean and above the mean value of the sum of scores in all domains, and poor if otherwise.

**Partograph use** was measured using chart review based on the time when a skilled birth attendant filled partograph during labor and childbirth. It was scored YES if the delivery assistant completed filling the partograph while the mother was laboring, NO if otherwise.

### Sample size determination and sampling technique

The sample size of the study was determined by using a single population proportion formula by assuming a 95% confidence interval, 5% margin error and taking 67.6% proportion of newborn care quality from a study done in Tigray located in Ethiopia [17].

$$ {\text{N }} = \frac{{({\text{Z}}\alpha /{\text{2}})^{{\text{2}}} ~ \times {\text{ p }}\left( {{\text{1}} - {\text{p}}} \right)}}{{{\text{d}}^{{\text{2}}} }} $$

Considering a 10% non-response rate, the final largest sample size obtained was 370 mother-newborn pairs. The facility audit was done in all functional health facilities.

A systematic random sampling technique was used to select the study subjects. At the onset, three districts (Dabat, Debark and Janamora) were randomly selected from eight. A systematic random sampling technique was employed to select the mothers, who deliver a live birth/s in all health centers and hospitals in the three districts. The sample size was proportionally allocated to health centers and hospitals based on the respective health facilities annual antenatal care attendance before this study.

### Data collection tool and method

An interviewer-administered structured questionnaire adapted from different kinds of literature and guidelines were employed to assess the newborn care implementation perceived quality [4, 17–25]. Facility audit assessment tools were used to collect data on the availability and readiness levels. Pre-test was done for data collection tools outside the study area. Information on socio-demographic, socio-economic obstetric and newborn care perceived quality-related characteristics were collected from mothers after delivery at health facilities. Data were collected by six trained first-degree midwives and nurses. The data collectors collected the information through a face to face interview of mothers in health facilities after delivery. The consistency and completeness of the data were regularly checked by supervisors (clinicians and researchers) throughout the entire data collection period.

Initially the questionnaire was prepared in English. This questionnaire was translated into the local language, Amharic, and back to English by translators who were bilingual and competent in the two languages to check the content validity.

### Data processing and analysis

The data were entered and cleaned using the Epi-Info version 7.1.5.0 software. Cleaning was made by running frequencies, proportions and summary statistics. The Principal Component Analysis (PCA) was employed to generate a wealth index. The composite index for each component, domain, and overall essential newborn care perceived quality was calculated by including all the question items in the respective category. The indices in each category were labeled as “Good” if the composite score was equal to the mean and above the mean, and “Poor” if otherwise. Then, these values were converted into dummy variables by assigning the value of “1” to “Good” and “0” to “Poor”. Inter-item consistencies between the variables were tested for the variables creating each component of the essential newborn care perceived quality using the Cronbach’s alpha (all variables were > 0.86). The cleaned data were exported to STATA version 14 software for analysis. Univariable logistic regression analysis was performed primarily to select variables for the final model based on *p*-value < 0.2. Hosmer–Lemeshow tests (*p* < 0.05) were used to measure the goodness of fit of the models. Multivariable binary logistic regression analysis was employed to control the possible effect of confounders and finally, the variables which had significant association were identified based on AOR with 95% CI and *p*-value ≤ 0.05.

## Results

### Characteristics of mothers and health facilities

A total of 338 mothers who delivered in 11 health facilities completed the interview with a response rate of 91.4%. The mean age of the participants was 26.4 years (SD = 5.7) with a minimum and maximum of 12 and 45 years respectively. The majority of mothers, 325 (96.2%) were married, 333 (98.5%) had no health insurance, 113 (33.4%) had poor household income, 314 (92.9%) were Christians, and 253 (74.9%) were housewives. Most mothers who numbered 124 (36.7%) were unable to read and write, and 185 (54.7%) of the participants used agriculture as a main source of income.

About 16 health facilities were agreed for a facility audit, but 5 of them had no deliveries during the data collection period due to security issues. Most health facilities were health centers 14 (87.5%). Basic emergency and essential obstetric and newborn care service readiness levels were poor among 9 (56.2%) health facilities and three-fourth of them had poor child immunization readiness levels. Nearly one-third of the health facilities had only one or two midwives (Table 1).

**Table 1:** Characteristics of health facilities and mothers in Northwest Ethiopia, 2019

Variable	Category	n (%)	95% CI (%)
A. Maternal characteristics (N = 338)			
Age group (years)	≤ 19	31 (9.2)	6.5, 12.8
	20–29	202 (59.8)	54.4, 64.9
	30–39	100 (29.6)	24.9, 34.7
	≥ 40	05 (1.5)	0.6, 3.5
Marital status	Married	325 (96.2)	93.5, 97.8
	Not married*	13 (3.8)	2.2, 6.5
Mothers occupation	Housewife	253 (74.9)	69.9, 79.2
	Merchant	24 (7.1)	4.8, 10.4
	Government employee	46 (13.6)	10.3, 17.7
	Others**	15 (4.4)	2.7, 7.2
Fathers occupation	Farmer	180 (53.3)	47.9, 58.5
	Merchant	53 (15.7)	12.2, 20.0
	Government employee	63 (18.6)	14.8, 23.2
	Private employ	13 (3.9)	2.2, 6.5
	Others +	29 (8.6)	6.0, 12.1
Religion	Christians	314 (92.9)	89.6, 95.2
	Muslim	24 (7.1)	4.8, 10.4
Family size (number)	1–3	126 (37.3)	32.3, 42.6
	≥ 4	212 (62.7)	57.4, 67.7
Residence	Urban	174 (51.5)	46.1, 56.8
	Rural	164 (48.5)	43.2, 53.9
Household income	Poor	113 (33.4)	28.6, 38.7
	Medium	112 (33.2)	28.3, 38.4
	Rich	113 (33.4)	28.6, 38.7
Source of household income	Agriculture	185 (54.7)	49.4, 60.0
	Monthly salary	77 (22.8)	18.6, 27.6
	Trade	45 (13.3)	10.1, 17.4
	Others***	31 (9.2)	6.5, 12.8
Health insurance	No	333 (98.5)	96.5, 99.4
	Yes	5 (1.5)	0.6, 3.5
B. Health facility characteristics (N = 16)			
District	Dabat	5 (31.3)	12.2, 59.8
	Debark	7 (43.8)	20.4, 70.2
	Janamora	4 (25)	8.6, 54.3
Health facility type	Health centers	14 (87.5)	57.0, 97.4
	Hospitals	2 (12.5)	2.6, 43.0
BEmEONC service readiness	Poor	9 (56.2)	29.8, 79.6
	Good	7 (43.8)	20.4, 70.2
CEmOC service readiness	Poor	12 (75)	45.7, 91.4
	Good	4 (25)	8.6, 54.3
Child immunization service readiness	Poor	6 (37.5)	16.1, 65.2
	Good	10 (62.5)	34.8, 83.9
Number of midwives	1 or 2	5 (31.3)	12.2, 59.8
	3 or 4	7 (43.8)	20.4, 70.2
	≥ 5	4 (25)	8.6, 54.3
Number of nurses	2–5	5 (31.3)	12.2, 59.8
	≥ 6	11 (68.8)	40.2, 87.8

*Single, divorced, separated and living together **Daily laborer, private employ and student

***Family support and daily labor,  +  = Daily laborer and student

### Obstetric characteristics of mothers

Almost a quarter of mothers which numbered 66 (19.5%) had their first pregnancy before 18 years of age. Mothers who had a prior history of stillbirths and neonatal deaths accounted for 29 (8.6%) and 27 (8%) respectively. The majority of mothers, 285 (84.3%), had attended antenatal care during pregnancy. Similarly, 323 (95.6) of the pregnancies were singleton, 285 (84.3%) of mothers had SVD mode of delivery, 295 (87.3%) deliveries were assisted by midwives and 187 (55.3%) deliveries were performed by male delivery assistants (Table 2).

**Table 2:** Obstetric characteristics of mothers and delivery assistants in selected health facilities, Northwest Ethiopia (N = 338), 2019

Variable	Category	n (%)	95% CI (%)
A. Pregnancy and delivery characteristics			
Age at first pregnancy/years/	Before 18	66 (19.5)	15.6, 24.1
	≥ 18	272 (80.5)	75.9, 84.4
Gestation age/weeks/	Preterm / ≤ 36/	48 (14.2)	10.9, 18.4
	Term /37–41/	284 (84.0)	79.7, 87.6
	Post-term / ≥ 42/	06 (1.8)	0.8, 3.9
Lifetime pregnancy	1–3 times	236 (69.8)	64.7, 74.5
	≥ 4 times	102 (30.2)	25.5, 35.3
Number of live children	0–3 children	256 (75.7)	70.9, 80.0
	≥ 4 children	82 (24.3)	20.0, 29.1
History of stillbirths	None	309 (91.4)	87.9, 94.0
	1 or 2 times	29 (8.6)	6.0, 12.1
History of spontaneous abortion	None	315 (93.2)	89.9, 95.4
	1 or 2 times	23 (6.8)	4.6, 10.1
History of neonatal death	None	311 (92)	88.6, 94.5
	1–3 times	27 (8)	5.5, 11.4
ANC attendance	No	53 (15.7)	12.2, 20.0
	Yes	285 (84.3)	80.0, 87.8
Health education by HEW	No	201 (59.5)	54.1, 64.6
	Yes	137 (40.5)	35.4, 45.9
Illness during the current pregnancy	No	244 (72.2)	67.1, 76.7
	Yes	94 (27.8)	23.3, 32.9
Type of pregnancy	Single	323 (95.6)	92.8, 97.3
	Twin or triplet	15 (4.4)	2.7, 7.3
Type of delivery	SVD	285 (84.3)	80.1, 87.8
	Instrumental	53 (15.7)	12.2, 20.0
B. Skilled birth attendant characteristics			
Profession	Medical Doctor	10 (3)	1.6, 5.4
	Nurse	14 (4.1)	2.5, 6.9
	Midwife	295 (87.3)	83.3, 90.4
	Health Officer	19 (5.6)	3.6, 8.7
Sex	Male	187 (55.3)	50.0, 60.6
	Female	151 (44.7)	39.4, 50.0
Washed hands before assisting the delivery	No	150 (44.4)	39.1, 49.7
	Yes	188 (55.6)	50.3, 60.9
Cleaned the perineum of the newborn	No	37 (10.9)	8.0, 14.8
	Yes	301 (89.1)	85.2, 92.0
Counseled on neonatal danger signs	No	159 (47)	41.7, 52.4
	Yes	179 (53)	47.6, 58.3

### Characteristics of newborns

The majority of newborns, 309 (91.4%) had a normal birth weight (2.5–4 kgs). About 94 (27.8%) neonatal illness cases were recorded. Most of the illnesses, 36 (38.3%) were observed on day 28 from birth, and 30 (31.9%) of them were recorded at birth. Fourteen neonatal illnesses ended with death and more than half, 54 (57.5%) of the ill neonates did not get healthcare (Table 3).

**Table 3:** Characteristics of newborns in selected health facilities, Northwest Ethiopia (N = 338), 2019

Variable	Category	n (%)	95% CI (%)
Birth weight	LBW/ < 2.5 kg/	24 (7.1)	4.8, 10.4
	NBW/2.5-4 kg/	309 (91.4)	87.9, 94.0
	Over/ ≥ 4 kg/	05 (1.5)	0.6, 3.5
Sex	Male	181 (53.6)	48.2, 58.8
	Female	157 (46.4)	41.2, 51.8
Stay in health facility after delivery	< 24 h	221 (65.4)	60.1, 70.3
	≥ 24 h	82 (24.3)	20.0, 29.1
	I do not know	35 (10.3)	7.5, 14.1
Neonatal illness (N = 94)	No	244 (72.2)	67.1, 76.7
	Yes	94 (27.8)	23.3, 32.9
Neonatal age at illness (N = 94) *(Each newborn had more than one illness and hence, the percentage adds up more than 100%)*	At birth	30 (31.9)	21.0, 29.5
	24 h from birth	10 (10.6)	6.9, 9.8
	7 days from birth	23 (24.5)	16.1, 22.6
	14 days from birth	21 (22.3)	14.7, 20.6
	28 days from birth	36 (38.3)	25.2, 35.4
Frequency of neonatal illness (N = 94)	One time	56 (59.6)	49.2, 69.2
	Two times	21 (22.3)	14.9, 32.1
	Three times	3 (3.2)	1.0, 9.6
	Death with illness	14 (14.9)	8.9, 23.8
Neonatal age at death (N = 14)	At birth	12 (85.7)	52.0, 97.1
	At 7th day	2 (14.3)	2.9, 48.0
Healthcare for ill neonates (N = 94)	No	54 (57.5)	47.1, 67.2
	Yes	40 (42.5)	32.8, 52.9

*

### Perceived quality of essential newborn care implementation

**Fig. 1:**
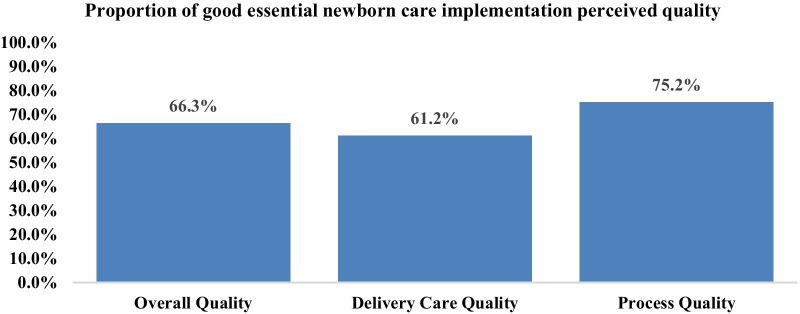
Essential newborn care quality among health facility deliveries in Northwest Ethiopia, 2019

This study revealed that the overall essential newborn care implementation perceived quality was 66.3% (224). The perceived quality provided during the delivery care domain was relatively worse with about 61.2% (207) poorer than the process perceived quality domain, which was 75.2% (254) (Fig. 1).

**Fig. 2:**
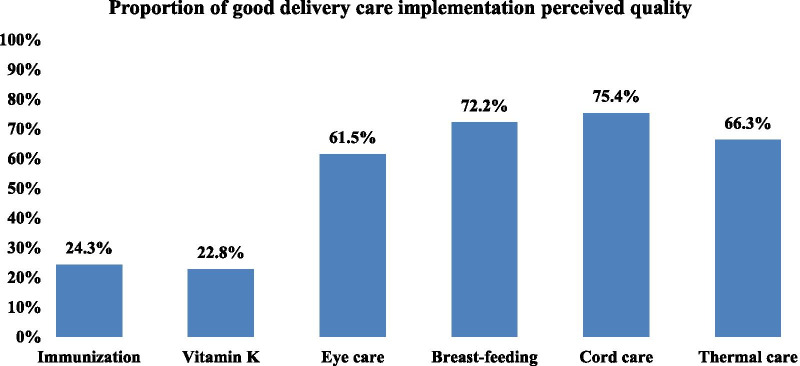
Delivery care quality among health facility deliveries in Northwest Ethiopia, 2019

**Fig. 3:**
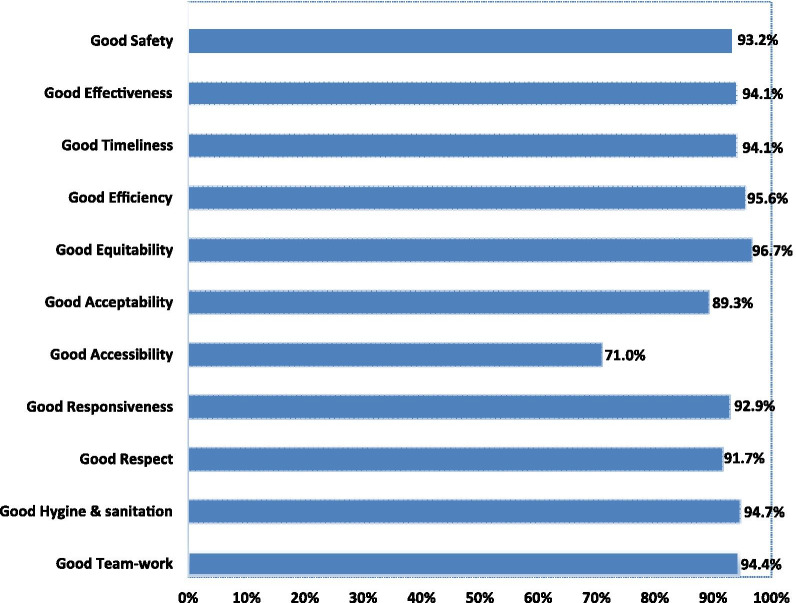
Process quality during delivery among health facility deliveries in Northwest Ethiopia, 2019

From the delivery care domain, components including cord care was 75.4% (255), breast-feeding was 72.2% (244), thermal care was 66.3% (224) and eye care was 61.5% (208). But, newborn immunization and vitamin K administration had the lowest perceived quality from delivery care domain components with 24.3% (82) and 22.8% (77), respectively. In relative terms accessibility, acceptability or people centeredness, respect for clients, responsiveness and safety were the components found to have had lower qualities with 71% (240), 89.3% (302), 91.7% (310), 92.9% (314) and 93.2% (315) respectively (Figs. 2 and 3).

### Predictors of essential newborn care implementation perceived quality

After controlling for facility, healthcare worker and newborn level characteristics; type of health facility, BEmEONC service readiness, child immunization service readiness, partograph use, birth weight, hypothermia, type of pregnancy, sex of the delivery assistant, friendly care during delivery, type of health facility and number of nurses in the health facility were the predictors that significantly affected the essential newborn care implementation perceived quality.

Mothers who got friendly care during delivery were five times (AOR = 5.1, 95% CI: 2.4, 11.0) more likely to have had good essential newborn care implementation perceived quality as compared to their counterparts. Healthcare workers who use partograph during delivery were three times (AOR = 3.0, 95% CI: 1.1, 8.6) more likely to have had good essential newborn care implementation perceived quality as compared to those healthcare workers who did not use partograph at all. Health facilities having good child immunization service readiness was three times (AOR = 2.9, 95% CI: 1.5, 5.7) more likely to have had good essential newborn care implementation perceived quality as compared to those facilities having poor child immunization service readiness levels. Newborns who did not face any illness after delivery were four times (AOR = 4.2, 95% CI: 1.6, 10.9) more likely to have had good essential newborn care implementation perceived quality as compared to those newborns who face hypothermia after delivery (Table 4).

**Table 4:** Predictors of essential newborn care quality among facility deliveries in Northwest Ethiopia (N = 338), 2019

Variable	Category	Newborn care quality		Odds ratio with 95%CI	
		Good (%)	Poor (%)	CoR	AoR
Type of health facility	Health centers	170 (75.9)	63 (55.3)	2.5 (1.6,4.1)*	2.8 (1.4, 5.8)**
	Hospitals	54 (24.1)	51 (44.7)	1	1
Number of nurses	Nine or less	74	25	1.8 (1.1, 2.9)*	1.7 (1.1, 2.9)*
	Ten or more	150	89	1	1
BEmEONC service readiness	Good	117	31	2.9 (1.8, 4.8)*	2.1 (1.2, 3.9)**
	Poor	107	83	1	1
Child immunization service readiness	Good	139	56	1.7 (1.1, 2.8)*	2.9 (1.5, 5.7)**
	Poor	85	58	1	1
Partograph use	Yes	27	5	3.0 (1.1, 7.9)*	3.0 (1.1, 8.6)*
	No	197	109	1	1
Birth weight /gram/	Low / < 2500/	23 (10.3)	26 (22.8)	1	1
	High / ≥ 2500/	201 (89.7)	88 (77.2)	2.6 (1.4, 4.8)*	1.9 (1.1, 3.6)*
Hypothermia	Yes	7	16	1	1
	Other illness	44	27	3.7 (1.4, 10.2)*	3.0 (1.1, 8.6)*
	No illness at all	173	71	5.6 (2.2, 14.1)*	4.2 (1.6, 10.9)**
Type of pregnancy	Singleton	219 (97.8)	104 (91.2)	4.2 (1.4,12.6)*	3.0 (1.1, 9.4)*
	Twin or triplet	5 (2.2)	10 (8.8)	1	1
Sex the delivery assistant	Female	84 (37.5)	67 (58.8)	1	1
	Male	140 (62.5)	47 (41.2)	2.4 (1.5,3.8)*	2.1 (1.3, 3.3)**
Friendly care during delivery	Yes	213 (95.1)	88 (77.2)	5.7 (2.7,12.1)*	5.1 (2.4, 11.0)**
	No	11 (4.9)	26 (22.8)	1	1

*p < .05, **p < .01, and ***p < .001

## Discussion

This study aimed to measure the magnitude and factors associated with essential newborn care implementation perceived quality among health facility deliveries in Northwest Ethiopia. Accordingly, only 66.3% of neonates received good implementation perceived quality of essential newborn care services. Despite the observed low implementation perceived quality of essential newborn care service readiness in the study area as compared to the national target, the study has observed and expressed room for improvement in contrast to the previous studies done in Ethiopia. Friendly care during delivery, partograph use, child immunization, and BEmEONC service readiness, having ≥ 2500 g birth weight, delivering in a health center, and facing no neonatal illness at all were the factors significantly associated with good essential newborn care implementation perceived quality.

The implementation perceived quality of essential newborn care services remains low in Ethiopia and Africa. The current study revealed that the overall essential newborn care implementation perceived quality was low (only 66.3%), which was comparable with other study results in Ethiopia [13, 14, 20, 21, 26, 27] and Africa [22, 23]. On the other hand, it was lower than the study findings in Ethiopia [17, 28, 29]. This difference could be due to a lack of comprehensiveness on the measurement tool for quality. The focuses of the previous studies were more on thermal care, cord care and/or breast-feeding components. Besides, the availability of BEmEONC service inputs (trained staff and guidelines, types of equipment, medicines and commodities) is not uniform across health facilities in different nations. The standardized procedure for providing essential newborn care is not commonly practiced. Therefore, in order to improve the essential newborn care implementation perceived quality and neonatal survival, the North Gondar administrative zone health office need to adhere with the essential newborn care policy by availing all the inputs of BEmEONC and child immunization services, provision of continuous training and motivation of healthcare workers for friendly care.

More specifically, the implementation perceived quality of thermal care was 66.3%, which was congruent with a study done in Wolaita Zone (65.3%) [30], higher than another study in Aksum Town (32.6%) [21], and lower than another study in Ethiopia and Bangladesh [24, 31]. Cord care implementation perceived quality was 75.4%, which was higher than the study findings in Ethiopia and Nepal [21, 24, 32], but lower than the 2016 demographic and health survey finding and other studies in Ethiopia and other developing countries [16, 30, 31, 33, 34]. The possible explanations for the difference might be due to variations in study settings (community/facility-based) and time of interview after delivery.

Breast-feeding implementation perceived quality was found to be 77.2%, which is higher than the 2016 and the 2019 demographic and health survey and a study finding in Ethiopia and other low-income countries [8, 16, 21, 24, 25, 30–35]. Newborn immunization implementation was 24.3%, which was low as compared to other delivery domain components. This finding is lower than a similar study done in Aksum Town (44.7%) and the 2016 and the 2019 Ethiopian demographic and health survey findings [8, 16, 21]. The variations might be due to the inadequate number of skilled and competent service providers, and variations in workload in the health facilities.

The perceived quality of essential newborn care implementation in health centers was nearly three times more likely to be good as compared to hospitals. This finding contradicts the expected general truth. Because, compared to periphery areas (health centers), the service given at hospitals is expected to be better in terms of perceived quality due to the availability of experienced and high-level human power, resource, and supplies. High workload of service providers at hospitals as a result of the high case follows, inadequate number of skilled providers, and poor job satisfaction could be the possible reasons for the poor hospital services perceived quality [36–38]. Therefore, it is important to give attention to the quality of care given in hospitals.

The mean readiness level of health facilities on BEmEONC tracer items was 43.8%, which is lower than the national average (68%) and higher than a study finding in Kenya (23.8%) [39]. Similarly, the health facilities’ readiness levels on child immunization services were 62.5, which was higher than the national average (54%) [40]. Consequently, health facilities having good BEmEONC service readiness were two times more likely to have good essential newborn care implementation perceived quality. Similarly, facilities having good child immunization service readiness were three times more likely to have good essential newborn care implementation perceived quality. This implies that the perceived implementation quality of essential newborn care service is highly dependent on the availability of inputs on trained staff, guidelines, equipment, medicines, and commodities.

This study revealed that those delivery assistants who used to fill the partograph were three times more likely to have good essential newborn care implementation perceived quality than those who did not use it. This finding is consistent with other studies in Tigray, Ethiopia, and Africa [17, 23, 38]. This could be attributed to poor attitude, knowledge, and skills on how to fill partograph among healthcare providers.

In the current study, mothers who received friendly care during delivery were five times more likely to receive good implementation perceived quality of essential newborn care. This finding is supported by a similar study in Tigray, Zimbabwe and developing countries [17, 41, 42]. This implies that there are mothers who neither received quality essential newborn care nor friendly care during delivery. The reasons could be due to poor skills and competency of service providers.

Neonates who did not face any illness were four times and those who face illness other than hypothermia were three times more likely to have good essential newborn care implementation qualities as compared to those neonates who face hypothermia. This is supported by a study finding in Lebanon that found a two-third reduction of hypothermia through the provision of good implementation perceived quality of essential newborn care [43]. Similarly, neonates having > 2500 g birth weight had two times more likely to have good essential newborn care implementation qualities. This indicates that quality of care is crucial for the betterment of neonatal outcomes. Consequently, the Ethiopian government and the North Gondar administrative zone health office in particular, need to adhere with the essential newborn care policy by availing the BEmEONC and child immunization service inputs, continuous training and motivation of healthcare workers for friendly care to improve the essential newborn care implementation perceived quality and neonatal survival.

Furthermore, the WHO, the United Nations Children Fund (UNICEF) and the United Nations Population Fund (UNFPA) should strengthen their efforts on improving the quality of the essential newborn care implementation during and immediately after birth through availing all the essential newborn care inputs in health facilities, and provision of tailored trainings and motivation of healthcare workers in order to improve newborn survival.

This study was undertaken with the following limitations. First, due to resource limitations, the authors could not include health facilities from multiple administrative zones of the country to generalize the findings at regional and national levels. Second, as all essential newborn care component are essential (mandatory), it is very difficult to measure and conclude ENC implementation perceived quality using mean value. Third, the balance of the response rates of some variables were low, which might affect the model fit the data reasonably well. Lastly, due to ethical concerns, data were collected through interviews, unlike observation, by using trained midwives and nurses from the mothers' perspective.

## Conclusions

This study revealed that the implementation perceived quality of newborn care was low because of poor readiness on BEmEONC and child immunization services, unfriendly care and none use of partograph. Friendly care during delivery, using partograph during delivery, BEmEONC service readiness, child immunization service readiness, delivering in a health center, having nine or less number of nurses, having a singleton pregnancy, having ≥ 2500 g birth weight, facing no neonatal illness, and having a male delivery assistant were the factors significantly associated with good essential newborn care implementation perceived quality. Hence, availing the BEmEONC and child immunization service inputs, continuous training and motivation of healthcare workers for friendly care are vital for improving essential newborn care implementation perceived quality in the study area and other similar settings.

## Data Availability

The dataset that supports the findings of this study is available from the corresponding author upon reasonable request.

## Abbreviations

ANC: Ante natal care
AOR: Adjusted odds ratio
BEmONC: Basic emergency and essential obstetric and newborn care
CEmOC: Comprehensive obstetric care
CI: Confidence interval
COR: Crude odds ratio
CSA: Central statistical agency
EMPIC: Enhanced management of pneumonia in the community
ENC: Essential newborn care
IRB: Institutional review board
LBW: Low birth weight
NBW: Normal birth weight
SD: Standard deviation
SVD: Spontaneous vaginal delivery
UoG: University of gondar
WHO: World Health Organization

## References

[1] Tunçalp Ö, Were W, MacLennan C (2015). Quality of care for pregnant women and newborns-the WHO vision BJOG. Int J Obstet Gynaecol.

[2] World Health Organization (2014). Every Newborn: an action plan to end preventable deaths.

[3] UNICEF (2012). Maternal and newborn health 2012.

[4] Coutin A. Essential Obstetric and Newborn Care: Practical guide for midwives, doctors with obstetrics training and health care personnel who deal with obstetric emergencies. Medecins Sans Frontieres. 2015. ISBN Number: 2-906498-98-X.

[5] WVI. Essential Newborn Care, 2016. http://www.wvi.org/health/intervention-2-essential-newborn-care. Accessed 10 Nov 2019.

[6] Malhotra S, Zodpey SP, Vidyasagaran AL, Sharma K, Raj SS, Neogi SB (2014). Assessment of essential newborn care services in secondary-level facilities from two districts of India. J Health Popul Nutr.

[7] UNICEF (2018). Every child alive: The urgent need to end newborn deaths.

[8] Ethiopia Demographic and Health Survey (EDHS). Key Indicators Report, Central Statistical Agency, Addis Ababa, Ethiopia, and The DHS Program, ICF Rockville, Maryland, USA, 2019.

[9] WHO, UNFPA, UNICEF, AMDD (2009). Monitoring emergency obstetric care a handbook.

[10] Yinger NV, Ransom E. Why invest in newborn health? In: Policy perspectives on newborn health. Washington, DC: Save the Children & Population Reference Bureau; 2003. p. 1–6.

[11] Darmstadt GL, Bhutta ZA, Cousens S, Adam T, Walker N (2005). Evidence-based, cost-effective interventions: how many newborn babies can we save?. Lancet.

[12] Essential Health Service Package of Ethiopia (2019). Ministry of Health.

[13] Tegene T, Andargie G, Nega A, Yimam K (2015). Newborn Care Practice and Associated Factors among Mothers who gave birth within one year in Mandura District Northwest Ethiopia. CMCH.

[14] Teshome K, Mekonen A, Genet D (2015). Community-based essential new born care practices and associated factors among women in the rural community of Awabel District, East Gojjam Zone, Amhara, Ethiopia, 2013. Int J Adv Sci Res.

[15] Tura G, Fantahun M, Worku A (2015). Neonatal care practice and factors affecting in Southwest Ethiopia: a mixed-methods study. BMC Int Health Hum Rights.

[16] Central Statistical Agency [Ethiopia] and ICF International: Ethiopia Demographic and Health Survey (2016). Addis Ababa, Ethiopia, and Calverton, Maryland.

[17] Girmatsion F, Yemane B, Alemayehu W (2019). Quality of intrapartum and newborn care in Tigray Northern Ethiopia. BMC Pregnancy Childbirth.

[18] Federal Democratic Republic of Ethiopia Ministry of Health (2018). Basic emergency obstetric & newborn care (BEmONC) training manual.

[19] Federal Ministry of Health of Ethiopia (2014). Neonatal intensive care unit (NICU) training, management protocol.

[20] Tayelgn A, Zegeye D, Kebede Y (2011). Mothers' satisfaction with referral hospital delivery service in Amhara Region, Ethiopia. BMC Pregnancy Childbirth.

[21] Berhe M, Medhaniye AA, Kahsay G, Birhane E, Abay M (2017). Essential neonatal care utilization and associated factors among mothers in public health facilities of Aksum Town, North Ethiopia, 2016. PLoS ONE.

[22] Owor MO, Matovu JKB, Murokora D, Wanyenze RK, Waiswa P (2016). Factors associated with adoption of beneficial newborn care practices in rural Eastern Uganda: a cross sectional study. BMC Pregnancy Childbirth.

[23] Nesbitt RC, Lohela TJ, Manu A, Vesel L, Okyere E, Edmond K, Owusu-Agyei S, Kirkwood BR, Gabrysch S (2013). Quality along the continuum: a health facility assessment of intrapartum and postnatal care in Ghana. PLoS ONE.

[24] Mersha A, Assefa N, Teji K, Shibiru S, Darghawth R, Bante A (2018). Essential newborn care practice and its predictors among mother who delivered within the past six months in Chencha District, Southern Ethiopia, 2017. PLoS ONE.

[25] Waiswa P, Peterson S, Tomson G, Pariyo GW (2010). Poor newborn care practices—a population based survey in Eastern Uganda. BMC Pregnancy Childbirth.

[26] Bereket Y, Mulat T, Wondimagegn P (2013). Mothers’ utilization of antenatal care and their satisfaction with delivery services in selected public health facilities of Wolaita Zone, Southern Ethiopia. Int J Sci Technol Res.

[27] Daba W, Lemma AM (2015). Assessment of magnitude and determinants of neonatal care practice among mothers in selected health Centers of Addis Ababa, Administration Ethiopia.

[28] Alemayehu K, Gurmessa T, Aderajew N, Getahun K (2016). Satisfaction with emergency obstetric and newborn care services among clients using public health facilities in Jimma Zone, Oromia Regional State, Ethiopia; a cross-sectional study. BMC Pregnancy Childbirth.

[29] Amdemichael R, Tafa M, Fekadu H (2014). Maternal satisfaction with the delivery services in Assela Hospital, Arsi zone Oromia region. Gynaecol Obstet.

[30] Chichiabellu TY, Baze M, Feleke HA, Birhanu WD, Antehun AA (2018). Essential newborn care practices and associated factors among home-delivered mothers in Damot pulasa Woreda, southern Ethiopia. Reprod Health.

[31] Islam MT, Nazrul I, Yukie Y, Monjura KN, Nawzia Y (2015). Newborn care practices in rural Bangladesh. RRN..

[32] Kaphle HP, Yadav DK, Neupane N, Sharma B (2013). Newborn care practices in rural communities of Nawalparasi District Nepal. JHAS.

[33] Saaka M, Iddrisu M (2014). Patterns and determinants of essential newborn care practices in rural areas of northern Ghana. Int J Popul Res.

[34] Adelaja LM (2011). A survey of home delivery and newborn care practices among women in a suburban area of Western Nigeria. ISRN Obstet Gynecol.

[35] Vijayalakshmi S, Patil R, Datta SS (2014). Community-based study on newborn care practices and its determinants in rural Pondicherry. India J Neonatal Biol.

[36] Hailu S, Enqueselassie F, Berhane Y (2009). Health facility-based maternal death audit in Tigray Ethiopia. Ethiopia J Health Dev.

[37] Wako G, Berhane Y (2000). Structural quality of reproductive health services in south-Central Ethiopia. Ethiop J Health Dev.

[38] Getachew A, Ricca J, Cantor D, Rawlins B, Rosen H, Tekleberhan A, et al: Quality of Care for Prevention and Management of Common Maternal and Newborn Complications: A Study of Ethiopia’s Hospitals, 2011, Maternal and Child Health Integrated Program (MCHIP) and USAID: Jhpiego Brown’s Wharf, 1615 Thames Street, Baltimore, Maryland 21231–3492, USA. https://www.mchip.net/sites/default/files/Ethiopia_QoC_formatted_final.pdf. Accessed 28 Dec 2019.

[39] Sum JT, Boibanda F, Ayuku D, Too KJ (2017). Assessing facility readiness to offer basic emergency obstetrics and neonatal care (BEmONC) services in health care facilities of west Pokot county Kenya. J Clin Simul Nurs.

[40] Ethiopian Public Health Institute (2016). Service Availability and Readiness Assessment (SARA Federal Ministry of Health and World Health Organization (WHO) 2016.

[41] Kanengoni B, Sari A, Eleanor H (2019). Women’s experiences of disrespectful and abusive maternal health care in a low resource rural setting in eastern Zimbabwe. Elsevier Midwifery.

[42] Freedman LP, Kruk ME (2014). Disrespect and abuse of women in childbirth: challenging the global quality and accountability agendas. Lancet.

[43] Andrews C, Whatley C, Smith M, Brayton EC, Simone S, Holmes AV (2018). Quality-improvement effort to reduce hypothermia among high-risk infants on a mother-infant unit. Pediatrics.

